# Bridging the Gap: A Case of Unique Anterior Segment Optical Coherence Tomography Presentation of Retrocorneal Membrane and Fibrous Bridging After Birth-Related Descemet's Membrane Detachment

**DOI:** 10.7759/cureus.103377

**Published:** 2026-02-10

**Authors:** Pravalika Rebbala, Siddharam S Janti, Sathwik N Reddy

**Affiliations:** 1 Ophthalmology, All India Institute of Medical Sciences, Bibinagar, Hyderabad, IND

**Keywords:** as-oct, birth trauma, descemet’s membrane detachment, forceps delivery, retrocorneal membrane

## Abstract

Descemet’s membrane detachment (DMD) is an uncommon corneal pathology that typically occurs following intraocular surgeries or direct ocular trauma. Congenital or birth trauma-related DMD is rare and often remains undiagnosed in asymptomatic individuals. Longstanding cases may develop fibrous bridging or retrocorneal membrane formation, making diagnosis challenging without advanced imaging.

We report the case of a 32-year-old asymptomatic male who presented for a routine ophthalmic evaluation. Slit-lamp examination of the right eye revealed a localized inferotemporal corneal opacity with subtle posterior corneal striae. The patient’s history was significant for a forceps-assisted birth complicated by right auricular deformity. Anterior segment optical coherence tomography (AS-OCT) demonstrated a planar DMD with fibrous bridging and retrocorneal membrane formation, sparing the visual axis. Best corrected visual acuity was 6/9 partial bilaterally, and intraocular pressures were normal. In the absence of symptoms or visual axis involvement, conservative management with regular follow-up was advised. At six months, the patient remained asymptomatic with stable findings.

Birth trauma-related DMD is rarely detected in adulthood, especially in the absence of visual impairment. The combination of external markers of birth trauma such as auricular deformity and characteristic posterior corneal striae should prompt consideration of this diagnosis. AS-OCT plays a crucial role in confirming and characterizing chronic DMD, particularly when slit-lamp findings are subtle. Previous studies have highlighted that these lesions may remain stable for years but can occasionally result in visually significant astigmatism or corneal decompensation, necessitating intervention.

To the best of our knowledge, this is the first report demonstrating fibrous bridging associated with birth trauma-related DMD using AS-OCT. This case underscores the importance of obtaining a thorough birth history and utilizing AS-OCT to evaluate unexplained posterior corneal changes. Conservative management is appropriate in asymptomatic cases without central corneal involvement, but long-term follow-up is essential to detect potential progression.

## Introduction

Descemet’s membrane is the basement membrane of corneal endothelial cells and plays a critical role in maintaining corneal transparency. Descemet’s membrane detachment (DMD) is most commonly encountered following intraocular surgery but may also occur due to blunt trauma, advanced age, or surgical technique-related factors [1].

Birth trauma-related DMD is rare and frequently underdiagnosed, particularly in asymptomatic adults. Excessive corneal stretching during forceps-assisted delivery can result in vertical or oblique tears of Descemet’s membrane [2]. These injuries may heal spontaneously or stabilize with minimal impact on corneal clarity, allowing them to remain clinically silent for years.

Honig classified obstetrical forceps-induced Descemet’s membrane tears into four histopathological types: Type I: Large tears with a fragment extending into the anterior chamber and scroll formation at the opposite end; Type II: Scrolls present at both margins of the tear; Type III: Small breaks healing by fibrosis at and posterior to the original tear; and Type IV: Small breaks with minimal fibrosis [3].

We report a unique case of chronic, asymptomatic DMD secondary to birth trauma that remained undetected for over three decades. Anterior segment optical coherence tomography (AS-OCT) revealed an unusual finding of fibrous bridging between a retrocorneal membrane and the posterior corneal surface, highlighting a rare sequela of perinatal corneal injury and the diagnostic value of AS-OCT in chronic DMD.

## Case presentation

**Figure 1:**
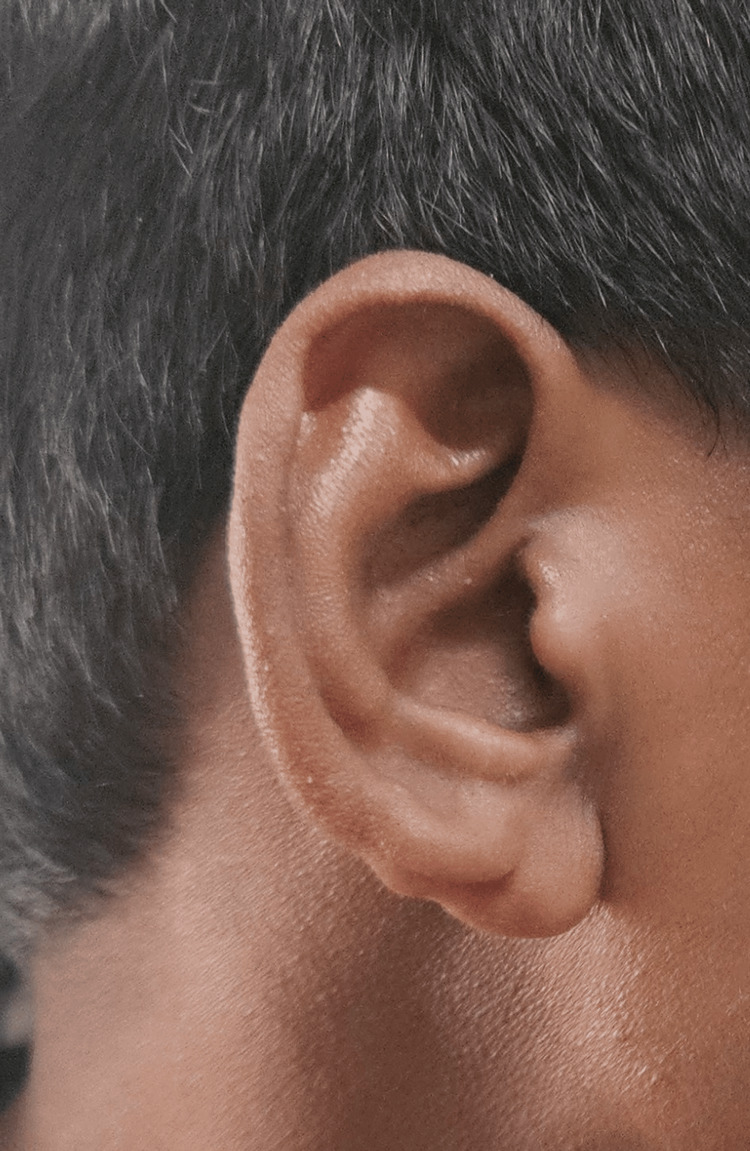
External examination revealed a deformity of the right pinna secondary to forceps induced birth trauma

A 32-year-old male presented to the ophthalmology outpatient department for routine evaluation. He was asymptomatic and denied any history of visual complaints, ocular pain, redness, trauma, or ocular surgery. There was no history of systemic illness. Notably, the patient had been delivered via forceps-assisted delivery and had residual deformity of the right pinna consistent with birth trauma (Figure 1).

Best-corrected visual acuity (BCVA) was 6/9 (partial) in both eyes on Snellen’s chart, with a refractive error of −2.75 diopters spherical. Near vision was N6 bilaterally. Intraocular pressures measured by non-contact tonometry were 14 mmHg in the right eye (OD) and 15 mmHg in the left eye (OS).

**Figure 2:**
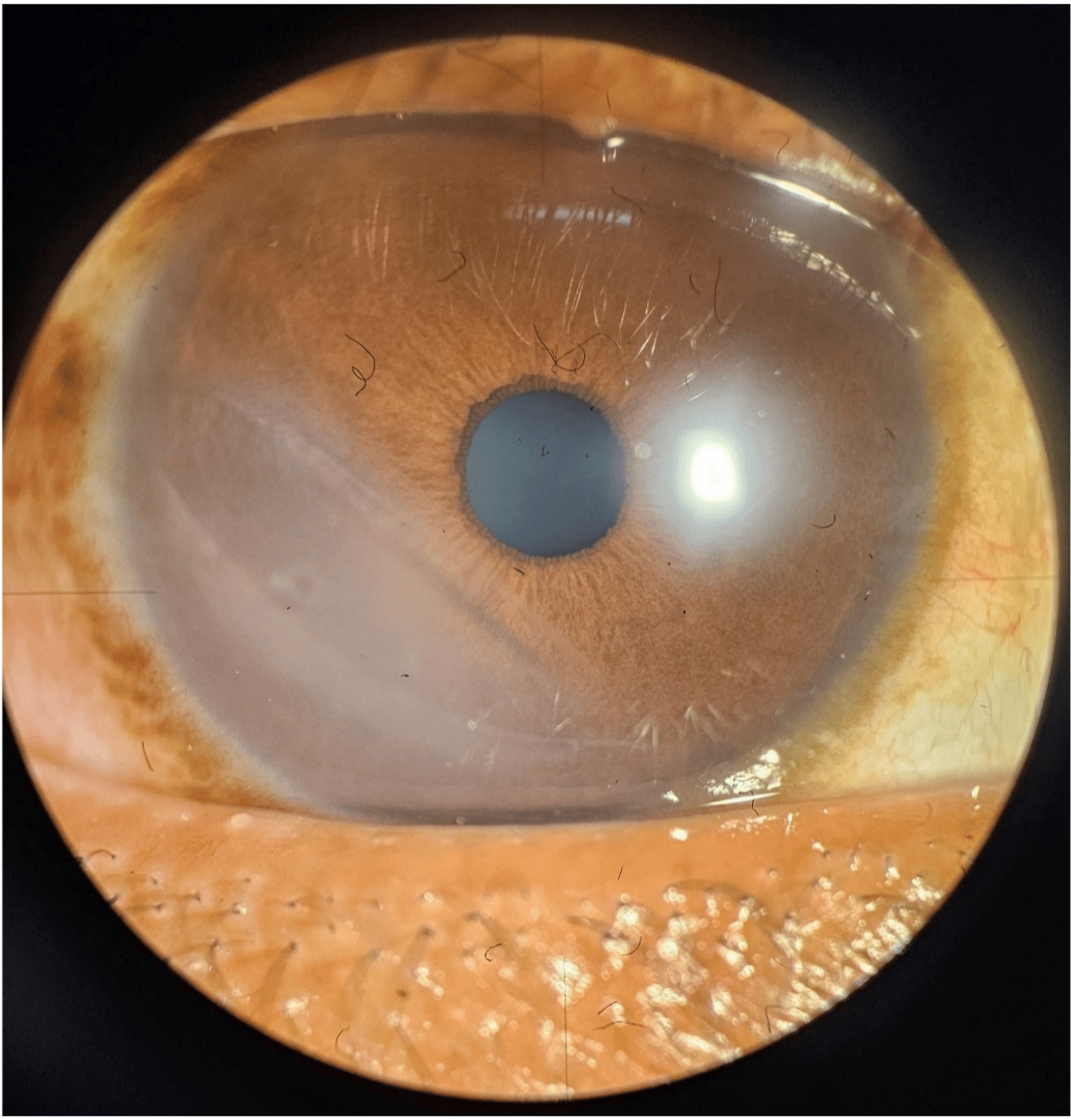
Diffuse illumination of right eye showing one small scarred bulla, mild opaque cornea in inferotemporal quadrant with hyperreflective bands placed obliquely in the posterior cornea surface sparing the pupil

Slit-lamp examination of the right eye revealed a localized inferotemporal corneal opacity with areas of scarred epithelial bullae, sparing the pupillary axis (Figure 2).

**Figure 3:**
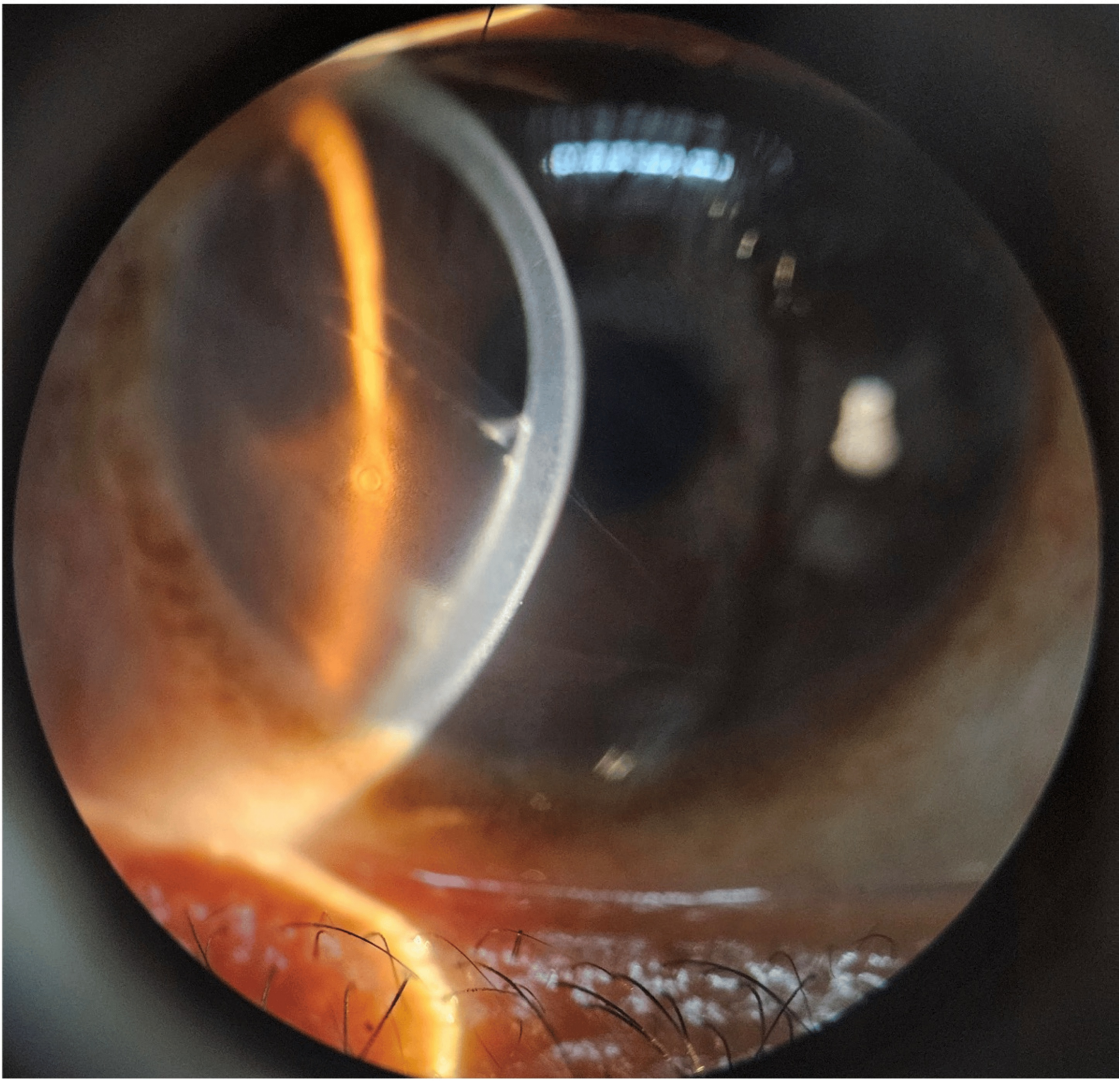
Slit-beam examination of the right eye revealed scarred epithelial bullae, mild stromal edema and two distinct, semi-translucent, parallel striae on the posterior corneal surface

Slit-beam examination showed mild underlying stromal edema and two semi-translucent, parallel posterior corneal striae suggestive of old Descemet’s membrane trauma (Figure 3). There was no evidence of active corneal edema. The anterior chamber was deep and quiet, with a clear crystalline lens. Examination of the left eye was unremarkable. Posterior segment evaluation revealed normal optic discs, maculae, and attached retinas in both eyes.

**Figure 4:**
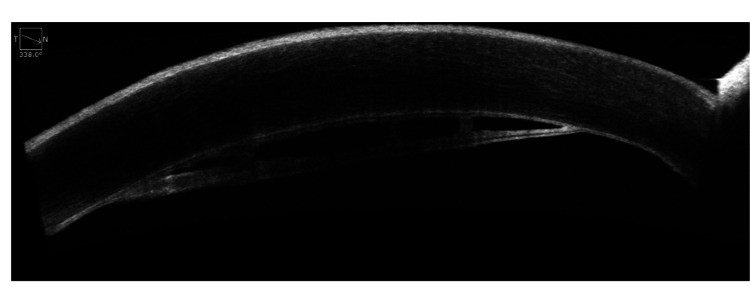
Anterior segment optical coherence tomography (AS-OCT) of the right eye demonstrated localized Descemet’s membrane detachment with fibrous bridging between the detached Descemet’s membrane and adjacent corneal endothelium

AS-OCT of the right eye demonstrated a localized planar DMD with a clear separation plane between the posterior stroma and the detached Descemet’s membrane. A retrocorneal membrane with distinct fibrous bridging extending between the detached membrane and the posterior corneal surface was noted (Figure 4). The detachment was confined to the inferotemporal quadrant and spared the visual axis.

Based on the clinical findings, imaging characteristics, and birth history, a diagnosis of chronic localized DMD secondary to forceps-related birth trauma was made. Given the patient’s lack of symptoms, stable visual acuity, and peripheral location of the lesion, conservative management with observation was advised.

At six-month follow-up, the patient remained asymptomatic. BCVA and slit-lamp findings were unchanged, and repeat AS-OCT showed no progression of the detachment or fibrous changes. Annual follow-up was recommended.

## Discussion

This case represents a rare, asymptomatic presentation of birth trauma-related DMD diagnosed incidentally in adulthood. Forceps-induced Descemet’s membrane tears occur when the globe is compressed between the orbital roof and the forceps blade, leading to transient elevation of intraocular pressure and horizontal stretching of the cornea. When the elastic limit of Descemet’s membrane is exceeded, vertical or oblique tears may result [2].

Corneal edema following such injuries may present in two phases: an early phase occurring shortly after birth and a delayed phase appearing decades later due to progressive endothelial decompensation [4,5]. The interval between injury and late-onset decompensation has been reported to range from 25 to 44 years [2]. In rare cases, the cornea may remain clear despite significant Descemet’s membrane damage [6].

In the present case, the lesion was peripheral and spared the visual axis, explaining the preserved visual acuity and absence of symptoms. Although many cases can be detected clinically, chronic or subtle DMD may be difficult to identify on slit-lamp examination alone. AS-OCT provides high-resolution, non-invasive imaging that allows precise delineation of the configuration, extent, and sequelae of DMD, including fibrosis and retrocorneal membrane formation [1].

The demonstration of fibrous bridging between the detached Descemet’s membrane and the posterior corneal surface represents a unique imaging finding and likely reflects chronic healing and stabilization following perinatal trauma. Management of DMD depends on symptomatology, location, and endothelial function. In asymptomatic cases with preserved vision and peripheral involvement, conservative observation is appropriate.

## Conclusions

This case highlights the critical role of meticulous history-taking, careful slit-lamp examination, and AS-OCT in identifying atypical corneal pathologies. Silent DMD with associated retrocorneal fibrosis presenting decades after birth-related trauma is rare and can be easily overlooked in asymptomatic individuals. Although visual function was preserved, long-term follow-up is warranted to monitor for age-related endothelial cell loss and potential endothelial decompensation.

The presence of external markers of perinatal trauma, such as auricular deformities, should prompt clinicians to obtain a detailed birth history and perform focused evaluation of the posterior cornea. AS-OCT is invaluable for accurately characterising chronic DMD and its long-term sequelae, including retrocorneal membrane formation and fibrous bridging. This report emphasizes the need for increased awareness among obstetricians regarding the possibility of ocular injury following assisted vaginal deliveries, the importance of documentation of traumatic deliveries, and the value of early ophthalmologic referral in the neonatal period. A coordinated multidisciplinary approach involving obstetricians, neonatologists, and ophthalmologists may facilitate early detection and prevent long-term visual morbidity, including amblyopia. Additionally, AS-OCT serves as a valuable diagnostic adjunct in identifying subtle Descemet’s membrane changes, such as fibrosis, which may not be evident on routine slit-lamp examination

This report documents fibrous bridging associated with birth trauma-related DMD using AS-OCT. Conservative management with long-term follow-up is appropriate in asymptomatic cases without visual axis involvement, with continued surveillance to detect potential late-onset corneal decompensation.
